# Influence of Grain Orientation and Grain Boundary Features on Local Stress State of Cu-8Al-11Mn Alloy Investigated Using Crystal Plasticity Finite Element Method

**DOI:** 10.3390/ma15196950

**Published:** 2022-10-07

**Authors:** Ce Zheng, Lijun Xu, Xiaohui Feng, Qiuyan Huang, Yingju Li, Zhongwu Zhang, Yuansheng Yang

**Affiliations:** 1 Shi-Changxu Innovation Center for Advanced Materials, Institute of Metal Research, Chinese Academy of Sciences, Shenyang 110016, China; 2 Key Laboratory of Superlight Materials and Surface Technology, Ministry of Education, College of Materials Science and Chemical Engineering, Harbin Engineering University, Harbin 150001, China

**Keywords:** Cu-Al-Mn alloy, crystal plasticity finite element method, local stress, grain boundary, deformation mechanism

## Abstract

Reducing the local stress in the vicinity of the grain boundaries is a favorable way to improve the super-elastic properties of super-elastic alloys. The crystal plasticity finite element method (CPFEM) was applied in this study to simulate the deformation behavior and local stress of a super-elastic Cu-8Al-11Mn (wt.%) alloy containing single grains with various orientations, columnar grains with different misorientation angles, and tri-crystals with distinct grain boundary morphologies. The results indicated that the stress distribution of single grains presented obvious orientation dependence during deformation. Uniformly distributed stress was observed in grains with orientations of 0° and 90° when more slip systems were activated during deformation. With the increase in the misorientation angles of columnar grains, the stresses in the vicinity of the grain boundaries increased, which was related to the difference in the shear stress of the slip systems in adjacent grains. When the difference in the shear stress of the slip systems in two adjacent grains was large, a local stress concentration formed in the vicinity of the grain boundary. Compared with the triple-junction grain boundaries, the local stresses of the straight and vertical grain boundaries were smaller, which was closely related to the number of activated slip systems on both sides of the grain boundary. The above results were obtained experimentally and could be used to design super-elastic alloys with high performance.

## 1. Introduction

Cu-based super-elastic alloys (SEAs) have the advantages of a low cost, good super-elastic (SE) properties, and excellent electrical and thermal conductivity, and they have broad application prospects for shock absorption and noise resistance in engineering structures, machinery, architecture, automobiles, high-speed rail, aerospace, and other fields [1,2]. Cu-based SEAs with a polycrystalline structure, however, lack sufficient super-elastic recovery strains and are too brittle to be cold-worked. The preparation of Cu-based super-elastic alloys with a low cost and high recovery strain has attracted increasing attention.

The super-elastic properties of SEAs are affected by orientation [3,4,5], misorientation angles [6], and the characteristics of the grain boundaries [7,8,9,10,11]. For instance, the super-elastic recovery strain of a Cu-Al-Ni alloy increased from 4% to 8.3% by changing the crystallographic orientations of single crystals [5]. The super-elastic behaviors of Cu-Al-Mn-Ni alloy sheets in different directions were studied by Sutou et al. [8]. The results showed that the super-elastic strain of the <001>-<102>//RD (rolling direction) orientation was larger (~10.3%), while the super-elastic strain of the <111> orientation was smaller (~2%), which was mainly related to the high stress generated in the grain. In addition to orientation and misorientation angles, the characteries of the grain boundaries have a great influence on the super-elastic recovery strain of Cu-based SEAs. Surprisingly, the super-elastic properties of bi-crystal structures with low-angle grain boundaries can be compared with those of single grains. The super-elastic properties of grain boundaries with different grain boundary characteristics (such as bamboo-like grains, columnar grains, and equiaxed grains) are obviously different. The bamboo crystal structure of Cu-Zn-Al and Cu-Al-Ni alloys shows excellent properties close to those of single crystals in terms of super-elasticity and fatigue resistance [9]. In addition to bamboo-like grains, a columnar Cu-Al-Mn alloy also showed excellent super-elastic properties (recovery strain of 10.1%) [10]. Liu et al. [11] compared the three microstructures of equiaxed grains, bamboo-like grains, and columnar grains and found that bamboo-like grains and columnar grains had better super-elastic properties than equiaxed grains, mainly because the grains had a better uniform deformation ability and low local stresses formed in the vicinity of the grain boundaries.

It is well-known that the super-elastic properties (such as the recovery strain) of SEAs mainly come from stress-induced martensitic transformation and its inverse transformation [12]. In order to achieve better super-elastic properties, a larger strain is required to induce martensitic transformation [13]. When grain deformation occurs with a relatively uniform stress distribution, martensitic transformation can only occur under large strains, thus stimulating the super-elastic behavior. If the stress distribution uniformity of the grains is poor, a stress concentration is easily formed during the deformation process; as a result, stress-induced martensitic transformation occurs under a low strain. Once a martensite lamella has formed, it proliferates rapidly, occupying the whole grain and preventing grains from further deformation, which limits the improvement of the super-elastic properties of SEAs. From above, it is evident that the distribution of stress in the matrix and at grain boundaries has a great deal of influence on the martensitic transformation and SE properties. Therefore, it is necessary to study the influence of the crystal orientations, misorientation angles, and grain boundary morphologies on the local stress distribution and deformation mechanism of Cu-based SEAs.

Until now, the local stress at the grain scale was difficult to measure and characterize by experiments, so numerical simulation methods were mostly used, which included molecular dynamics [14] and the crystal plasticity finite element method [15,16,17]. As we all know, molecular dynamics is used to study and analyze the physical movement of atoms and molecules, which plays an important role in understanding the microscopic mechanism of the plastic deformation of materials. However, the computational scale of molecular dynamics is too small (usually the nanoscale), making it difficult to predict the mechanical behavior on the macroscale. The crystal plasticity finite element method (CPFEM) can be used to investigate the mechanical properties of materials on a scale ranging from micro single crystals to macro parts. In this paper, from the point of view of stress formation, theoretical guidance is provided to improve the super-elastic properties of a Cu-Al-Mn alloy. Therefore, CPFEM was more suitable for studying the stress formation mechanism between grains. Until now, CPFEM has been widely used to predict grain orientation rotation [18,19,20], the inhomogeneity of plastic strain and local stress [21,22], and grain orientation distribution [23,24,25] during deformation. A systematical evaluation of the effect of grain orientations and grain boundary features on the local stress state of a Cu-Al-Mn alloy using CPFEM will provide theoretical guidance for understanding the impact of grain features on SE properties at the grain scale.

In this paper, the crystal plastic finite element method (CPFEM) was applied to analyze the local stress state of a Cu-8Al-11Mn alloy with different crystallographic orientations, misorientation angles, and morphologies, and the deformation mechanism of local stress was systematically investigated.

## 2. Crystal Plasticity Theory and Modeling

### 2.1. Crystal Plasticity Theory

#### 2.1.1. Deformation Configuration

**Figure 1:**
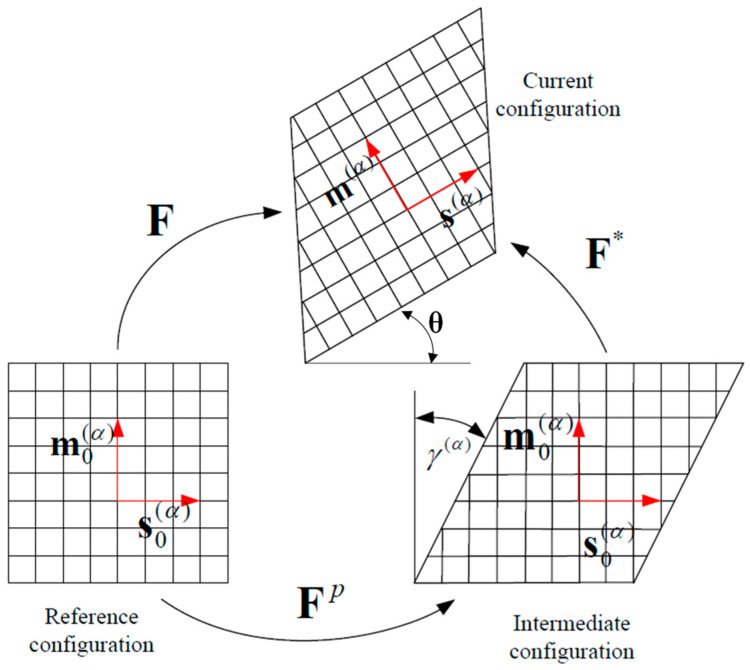
Kinematic diagram of crystal elastoplastic deformation [26].

Crystal deformation is caused by lattice distortion and rotation due to dislocation sliding along a slip direction on a slip plane. A single dislocation slip produces displacement discontinuity in the grain, which is difficult to express by a continuous deformation gradient. However, due to the large number of dislocations in the grain, from the mesoscopic point of view, it can be assumed that the slip is uniform in the crystal, and the whole crystal can be regarded as a continuous medium, so dislocation slip can be described by the field variable deformation gradient tensor of the continuous medium. Lattice distortion can be regarded as the elastic deformation of the continuous medium and can be approached with the method of elasticity. The deformation of grains under an external load consists of two parts: the elastic deformation caused by lattice distortion and rotation, and the plastic deformation caused by dislocation sliding along the slip system. These two components occur simultaneously during grain deformation. For the convenience of mathematical description, crystal deformation can be divided into two steps: first, the plastic deformation caused by the dislocation slip; second, the elastic deformation produced by lattice distortion on the basis of plastic deformation. Therefore, it is necessary to add an intermediate configuration between the pre-crystal-deformation reference configuration and the current post-deformation configuration to describe the whole deformation process of the grain, as shown in Figure 1 [26].

According to the continuum theory, the total deformation gradient from the reference configuration to the current configuration is:

(1) $$\;F=\frac{\partial x}{\partial X}$$

where ***F*** is the deformation gradient, ***x*** is the position coordinate of the material point in the current configuration, and ***X*** is the position coordinate of the material point in the reference configuration. The total deformation gradient can be decomposed into the deformation gradient caused by crystal slip according to the above analysis ***F****^P^* and the elastic deformation gradient caused by lattice deformation and the rigid rotation of the crystal F*:

***F*** = F* ·***F****^P^* (2)

From the lattice in Figure 1, it can be assumed that the material particles in the crystal are embedded in the lattice and are deformed by shear sliding and rotation along with the lattice. A slip system (*α*) in a crystal is expressed by the normal vector $m_{0}^{\left(\alpha \right)}$ of the slip plane and the directional vector $s_{0}^{\left(\alpha \right)}$ along the slip direction of slip system *α*, which are unit vectors and satisfy the orthogonal relation:

(3) $$m_{0}^{\left(\alpha \right)}\times s_{0}^{\left(\alpha \right)}=0$$

The plastic deformation gradient tensor is expressed as:

(4) $${\dot{\;F}}^{P}{\left(F^{P}\right)}^{-1}=\overset{N}{\underset{\alpha =1}{\sum }}{\dot{\gamma }}^{\alpha }\left(m_{0}^{\left(\alpha \right)}⊗s_{0}^{\left(\alpha \right)}\right)$$

where ${\dot{\gamma }}^{\alpha }$ represents the shear strain rate of the slip system *α*, *N* is the total number of slip systems, $m_{0}^{\left(\alpha \right)}$ is the original normal vector of the slip plane, and $s_{0}^{\left(\alpha \right)}$ is the original directional vector of the slip direction in the reference configuration.

#### 2.1.2. Rate-Dependent Deformation Kinetic and Hardening Model

The rate-dependent hardening model based on an exponential function proposed by Asaro and Needlem [27] was used in this investigation. The relationship between the resolved shear stress *τ^α^* and the shear strain rate ${\dot{\gamma }}^{\alpha }$ of each slip system was modeled. The shear strain rate ${\dot{\gamma }}^{\alpha }$ is expressed by Equation (5):

(5) $${\dot{\gamma }}^{\alpha }={\dot{\gamma }}_{0}^{\alpha }\mathrm{sgn}\left(\tau^{\alpha }\right){\left|\frac{\tau^{\alpha }}{g^{\alpha }}\right|}^{n}$$

(6) $$\tau^{\alpha }=m^{\alpha }\cdot \sigma \cdot s^{\alpha }$$

where ${\dot{\gamma }}_{0}^{\alpha }$ is a reference shear strain rate of slip system *α*; *n* is the strain rate sensitivity coefficient; *g^α^* is the current strength of slip system *α*; $\tau^{\alpha }$ is the resolved shear stress of slip system *α*; σ is the Cauchy stress tensor; and $m^{\alpha }$ and $s^{\alpha }$ are the normal vector of the slip plane and the directional vector of the slip direction in the current configuration, respectively.

$g^{\alpha }$ is related to the shear strain, which was determined by Equations (7)–(9):

(7) $$g^{\alpha }=\overset{N}{\underset{\beta =1}{\sum }}h_{\alpha \beta }\left|{\dot{\gamma }}^{\beta }\right|$$

(8) $$h_{\alpha \beta }=q_{\alpha \beta }h_{\alpha \alpha }$$

(9) $$h_{\alpha \alpha }=h_{0}sech^{2}\left|\frac{h_{0}\gamma }{\tau_{s}-\tau_{0}}\right|$$

where *h_αβ_* is the hardening coefficient when *α* = *β*; *h_αβ_* is the self-hardening coefficient *h_αα_*, when *α* ≠ *β*; *h_αβ_* is the latent hardening coefficient; *q_αβ_* is the scale factor; *h*_0_ is the initial hardening modulus; *τ_s_* is the saturated shear stress; *τ*_0_ is the initial critical shear stress; and *γ* is the cumulative shear strain on all slip systems.

(10) $$\gamma =\int_{0}^{t}\left|{\dot{\gamma }}^{\alpha }\right|dt$$

#### 2.1.3. Plastic Constitutive Relationship of Single Crystal

The elastic properties during deformation are not affected by plastic deformation, so the following elastic constitutive equation could be adopted:

(11) σ∇e=L:De

where σ∇e is the Jaumann derivative of the Kirchhoff stress tensor with the intermediate configuration as the reference state, ***L*** is the instantaneous elastic modulus tensor, and ***D****^e^* is the elastic part of the deformation rate tensor.

Furthermore, the plastic constitutive relation of a single crystal can be described as:

(12) σ∇=L:D−∑α=1N(L:Pα+βα)γ˙α

where σ∇ is the Jaumann derivative of the Cauchy stress tensor with the initial configuration as the reference state; ***D*** is the strain rate tensor; $P^{\alpha }$ and $\;\beta^{\alpha }$ are expressed as (13) and (14), respectively; $P^{\alpha }$ and ***W***^α^ are the symmetric and antisymmetric sections of the Schmid factor, respectively; and ***σ*** is the Cauchy stress tensor.

(13) $$P^{\alpha }=\frac{1}{2}\left(s^{\alpha }⊗m^{\alpha }+m^{\alpha }⊗s^{\alpha }\right)$$

(14) $$\beta^{\alpha }=W^{\alpha }\sigma -\sigma W^{\alpha }$$

(15) $$W^{\alpha }=\frac{1}{2}\left(s^{\alpha }⊗m^{\alpha }-m^{\alpha }⊗s^{\alpha }\right)$$

### 2.2. Crystal Plasticity Modeling

Cu-8Al-11Mn (wt.%) alloys have a high anisotropy coefficient of elastic constants, i.e., C_11_ = 139.329 GPa, C_12_ = 115.868 GPa, and C_44_ = 102.718 GPa. The lattice stability criterion should be satisfied, whereby C_11_ − C_12_ > 0, C_11_ + 2C_12_ > 0, and C_44_ > 0. The Poisson’s ratio constant of μ is 0.336. The β phase of Cu_2_AlMn is a L21-type superlattice structure, whose main slip system is {111} <110>, which is a face-centered cubic (FCC) crystal system. Detailed information regarding the slip systems is shown in Table 1.

**Figure 2:**
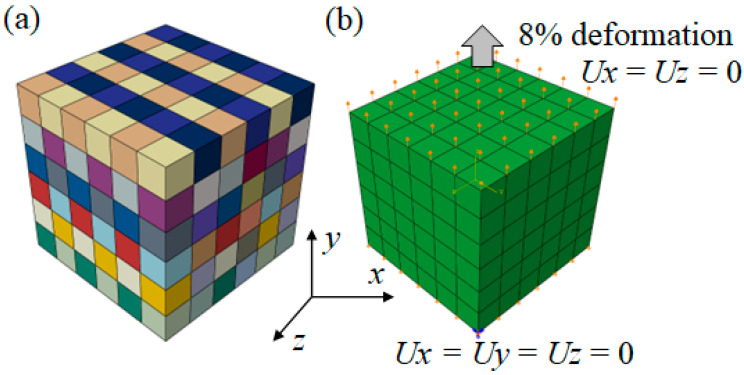
CPFEM model: (**a**) geometric model; (**b**) boundary conditions.

In order to determine the hardening parameters of the slip systems, a cube with a size of 6 mm × 6 mm × 6 mm was built, as shown in Figure 2a. Figure 2b shows a schematic diagram of the boundary conditions set by the CPFEM. A tensile displacement in the Y direction on the upper surface of the cube was applied with a deformation of 8%. The displacement in the X and Z directions was set to 0. In addition, the displacement in the X, Y, and Z directions on the bottom surface of the cube was set to 0. The cube was meshed by C3D8R elements with a size of 1 mm × 1 mm × 1 mm. Each element represented one grain with a random orientation. The step time was 0.001, and the time period was 1.

**Figure 3:**
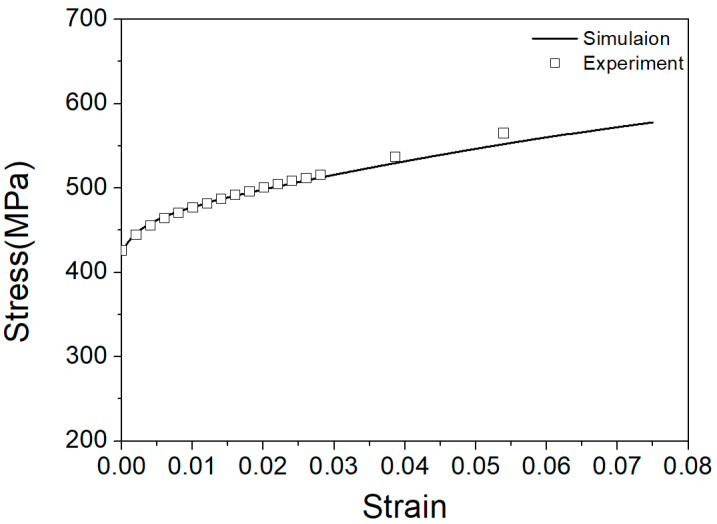
Stress-strain curves under tension (squares - experimental results, line - simulation results).

The hardening parameters of the slip systems of the Cu-8Al-11Mn alloy are shown in Table 2. The strain rate was 0.001 s^−1^, and the ratio of self-hardening and latent-hardening parameters was 1. The results of the simulation and experiment are shown in Figure 3, which highlights small discrepancies (a maximum of 3%) between the two sets of findings. In conclusion, these hardening parameters could be used to describe the harden behavior of a Cu-8Al-11Mn alloy under tension.

**Figure 4:**
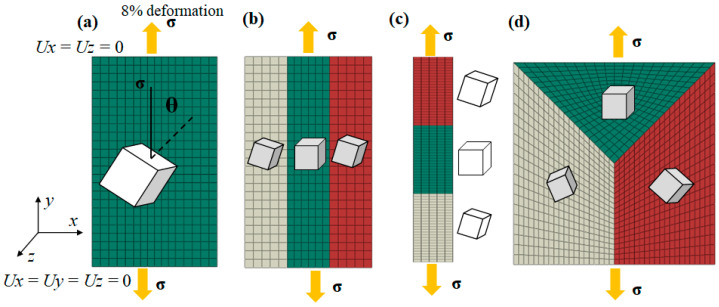
Geometric model and meshes of (**a**) single grains (SGs), (**b**) columnar grains (CGs), (**c**) bamboo-like grains (BLGs), and (**d**) equiaxed grains (EGs).

Based on the material parameters in Table 2, single-grain (SG), columnar-grain (CG), bamboo-like-grain (BLG), and equiaxed-grain (EG) models were established, as shown in Figure 4. The geometric dimensions of each model were 30 mm (x) × 50 mm (y) × 2 mm (z) for single grains; 10 mm (x) × 50 mm (y) × 2 mm (z) for columnar grains and bamboo-like grains; and 40 mm (x) × 40 mm (y) × 2 mm (z) for equiaxed grains. The single-grain models contained only one orientation, while the columnar-grain, bamboo-like-grain, and equiaxed-grain models contained three orientations. The angle between the (001) plane and loading direction ***σ*** was defined as θ, which ranged from 0° to 90° at a step of 15° to represent single grains with distinct orientations. In order to study the effect of the misorientation angles of adjacent grains on local stress, a plastic geometric model of columnar grains with misorientation angles of 5°, 10°, 15°, 30°, 45°, and 60° was established. That is, both low-angle grain boundaries (5°–15°) and high-angle grain boundaries (15°–60°) were considered in the models. These models were meshed by C3D8R elements with a size of 2 mm × 2 mm × 2 mm. A tensile displacement in the Y direction on the upper surface of the single-crystal, columnar-grain, bamboo-like-grain, and equiaxed-grain models was applied with a deformation of 8%. The displacement in the X and Z directions was set to 0. Meanwhile, the displacement in the X, Y, and Z directions on the bottom surface of the cube was set to 0. The step time was 0.001, and the time period was 1.

## 3. Results

### 3.1. Effect of Grain Orientation on Stress Distribution in Columnar Single Crystals

**Figure 5:**
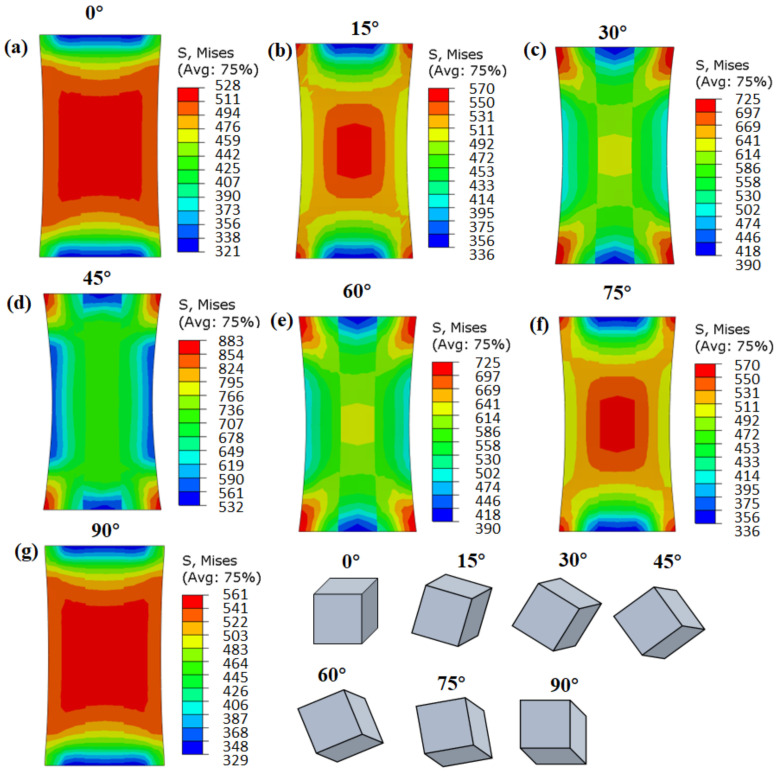
CPFEM simulation results of effective stress for columnar single crystals with different grain orientations: (**a**) 5°, (**b**) 15°, (**c**) 30°, (**d**) 45°, (**e**) 60°, (**f**) 75°, and (**g**) 90°.

Figure 5 shows the stress distribution of columnar single crystals with different orientations after tensile deformation. The stress distribution inside grains with orientations of 0° and 90° was relatively uniform, without an obvious stress concentration area (Figure 5a,g). The stress distribution in the middle of the 15 °, 30°, 45°, 60°, and 75° grains evidenced a high-stress region in the center and a low-stress region at the edge (Figure 5b–f), though with the same boundary conditions. In essence, distinct stress distribution patterns formed in single crystals with different grain orientations. However, there were high-stress regions on the top and bottom sides of all the single grains due to the fixed boundary conditions. The above results showed that the deformation of grains was uniform, and local stress concentrations were not easily produced when the grains were orientated at angles of 0° and 90°.

**Figure 6:**
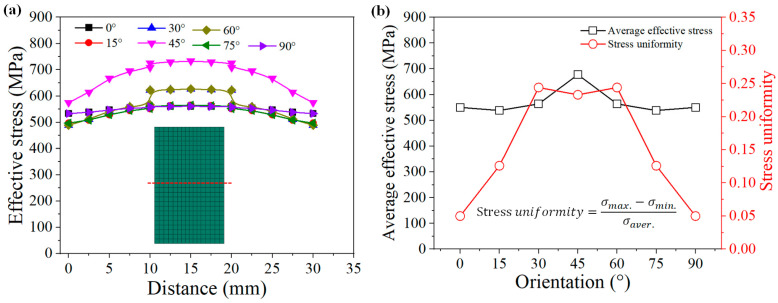
Stress distribution along red trace line for different grain orientations: (**a**) stress distribution curves; (**b**) average effective stress and stress uniformity.

A tracking path was inserted into the middle of the single crystals, and the results are shown in Figure 6a. From the left edge (x = 0 mm) to the right edge (x = 30 mm) of the models, the effective stresses first increased and then decreased, reaching the maximum value in the middle (x = 15 mm). The maximum effective stress for the 0°, 15°, 30°, 45°, 60°, 75°, and 90° grains was 560 MPa, 565 MPa, 624 MPa, 733 MPa, 624 MPa, 565 MPa, and 560 MPa, respectively. The effective stress increased from 0 ° to 45 ° and then decreased from 45° to 90°. Furthermore, the stress uniformities were calculated according to Equation (16), as shown in Figure 6b. The stress uniformity of the 0° and 90° grains was the lowest (about 0.05), and they also had a lower effective stress. In contrast, the deformation of the 15°, 30°, 45°, 60°, and 75° grains was uneven, with larger stress uniformity.

(16) $$Stress\;uniformity=\frac{\sigma_{max.}-\sigma_{min.}}{\sigma_{aver.}}$$

### 3.2. Effect of Misorientation Angles on Local Stress Distribution of Columnar Grains

**Figure 7:**
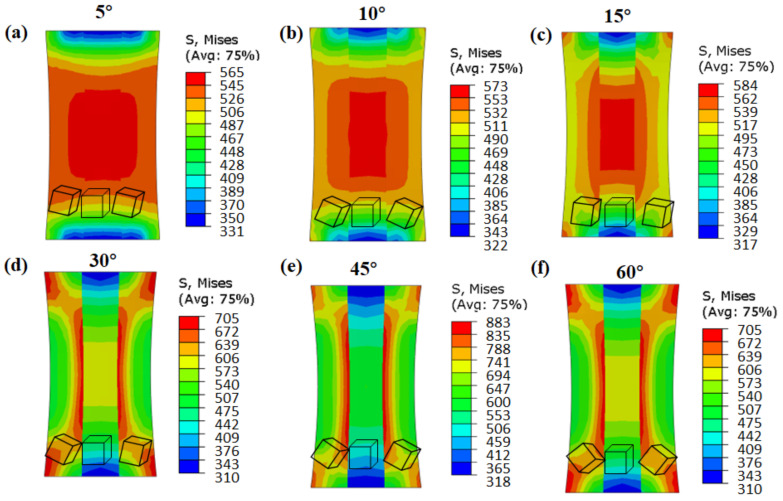
CPFEM simulation results of effective stress for columnar grains with different misorientation angles: (**a**) 5°, (**b**) 10°, (**c**) 15°, (**d**) 30°, (**e**) 45°, and (**f**) 60°.

Figure 7 shows the local stress distribution of columnar grains with different misorientation angles. It can be seen that the stress distribution near the low-angle grain boundaries (Figure 7a–c: 5°, 10°, and 15°) was uniform, and there was no stress concentration area, with a small stress value of 560~580 MPa. However, near the high-angle grain boundaries (Figure 7d–f: 30°, 45°, and 60°), there were obvious stress concentration areas, with gradient distribution features from the grain boundary to the interior of the matrix. 

**Figure 8:**
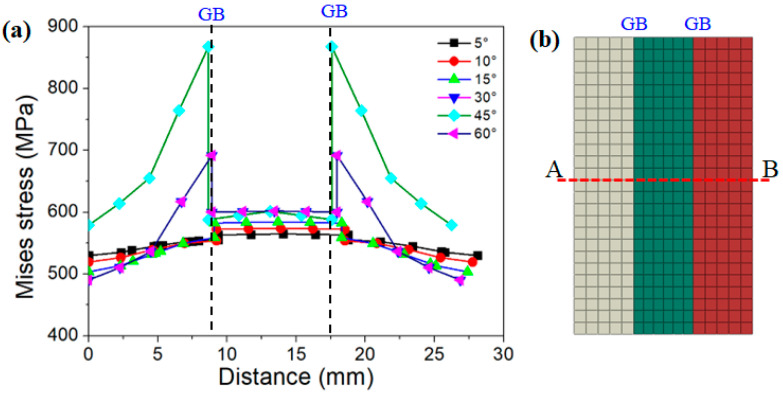
Stress distribution along trace line (A-B) of columnar grains with different misorientation angles: (**a**) stress distribution curves; (**b**) selected trace line A-B.

In order to show the effective stress distribution inside the grain and in the vicinity of the grain boundary more clearly, a tracking path (A-B) was inserted, which passed through the middle of the three columnar grains, as shown in Figure 8b. Figure 8a indicates the distribution of effective stress in columnar grains with different misorientation angles. There was a clear stress peak area in the grain boundary (GB). With the increase in the distance from the grain boundary, the stress value gradually decreased, indicating that the stress concentration was maximum at the GB. Comparing the results of six misorientation angles, it could be seen that the stress values of the columnar grains near the grain boundaries with low misorientation angles (5°, 10°, and 15°) did not differ substantially from the stress values of the matrix (difference of about 100 MPa). However, the stresses near the grain boundaries with high misorientation angles (30°, 45°, and 60°) were much greater than the stresses inside the grain (difference of about 350 MPa).

### 3.3. Effect of Grain Boundary Morphology on Local Stress Distribution

**Figure 9:**
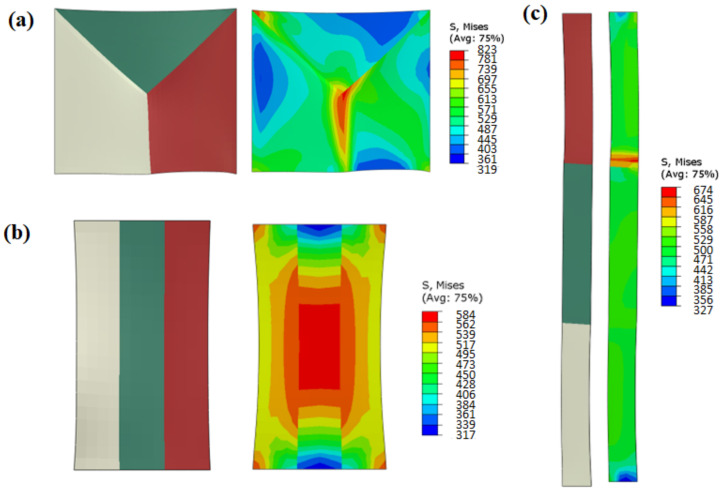
Effective stress of different grain boundary morphologies: (**a**) triple-junction GB of EG; (**b**) parallel GB of CG; (**c**) transverse GB of BLG.

Figure 9 shows the effective stress simulation results of grains with different grain boundary morphologies. The grain boundaries of the EGs were triple-junction GBs, those of the BLGs were transverse GBs, and those of the CGs were parallel GBs. There was an obvious stress concentration near the triple-junction GBs of the equiaxed grain structure (see the right-hand side of Figure 9a), with a large value of 823 MPa. There was no obvious stress concentration near the parallel GBs of the CGs, with the uniform stress distribution as shown on the right-hand side of Figure 9b, and the maximum stress value was 584 MPa. In the transverse GBs of the BLGs, the stress concentration area was only observed near the grain boundary of the upper and middle grains (red grain and green grain), as displayed on the right-hand side of Figure 9c, but the stress value was small (about 617 MPa).

**Figure 10:**
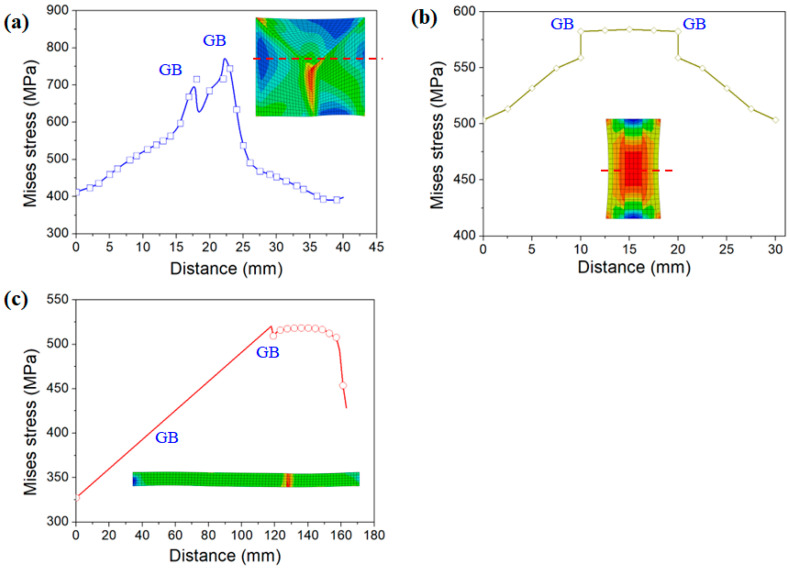
Effective stress curves of different grain boundary morphologies: (**a**) triple-junction GBs of EGs; (**b**) parallel GBs of CGs; (**c**) transverse GBs of BLGs.

Figure 10 shows the distribution curve of effective stress for the three grain morphologies. The effective stress value near the triple-junction GBs was large (at the distance of 17 mm and 22 mm in Figure 10a), and the stress value gradually decreased from the grain boundary to the grain interior (0–15 mm and 25–40 mm in Figure 10a). For the parallel GBs of the CGs, the stress distribution was more uniform from the grain boundary to the grain interior (Figure 10b). For the transverse GBs of the BLGs, almost no stress concentration area was observed in the two grains on the left. An obvious high-stress area was observed at the 130 mm position on the right grain boundary (Figure 10c), where the stress value was only 500 MPa, which was the smallest of the three microstructures.

## 4. Discussion

### 4.1. Effect of Grain Orientation on Deformation Mechanism of Columnar Single Crystals

**Figure 11:**
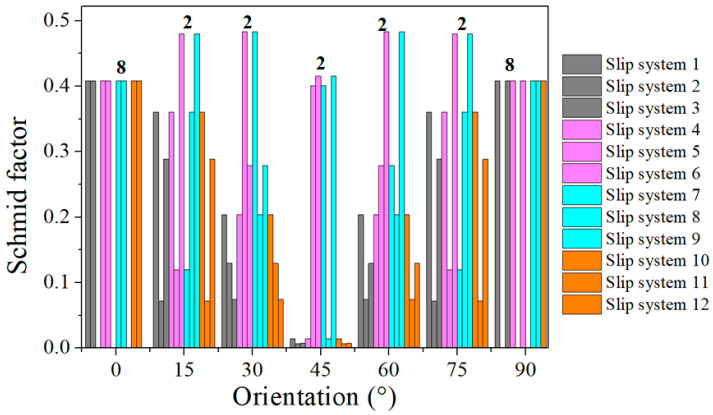
Calculation results of Schmid factors for slip systems of single grains with different orientations.

It is well known that the stress during grain deformation is closely related to the activated slip systems, which are considerably affected by the grain orientation and loading direction. In order to describe the relationship between orientation and loading direction, the Schmid factor (SF) values of the slip systems for each oriented grain were calculated, as shown in Figure 11. The slip systems with maximum SF values were called equivalent initial slip systems (EISSs) [28]. According to the statistics, the number of EISSs of each orientation were eight for 0° grains, two for 15° grains, two for 30° grains, two for 45° grains, two for 60° grains, two for 75° grains, and eight for 90° grains. In FCC materials, the more activated slip systems, the stronger the coordinated deformation ability of the grains. That is, when a certain slip system is difficult to activate due to low SF values, other slip systems can be activated to coordinate deformation. When the number of activated slip system is small, only a small number of slip systems coordinate deformation [29]. Zhao et al. [28] investigated the characteristics of stress distribution inside single grains with different orientations in an FCC aluminum alloy and found that the greater the number of EISSs during deformation, the more symmetrical and uniform the stress distribution inside the grains.

In addition, the activated slip systems of each grain orientation during tensile deformation were calculated. The relative activity of the slip system was calculated as shown in Equation (17).

(17) $$R_{t}^{\left(\alpha \right)}=\frac{\Delta \gamma_{t}^{\left(\alpha \right)}}{\sum_{\alpha =1}^{n}\Delta \gamma_{t}^{\left(\alpha \right)}}$$

where $R_{t}^{\left(\alpha \right)}$ is the relative activity of the total strain of slip system *α* at time *t*, $\Delta \gamma_{t}^{\left(\alpha \right)}$ is the shear strain of the slip system *α* at time *t*, and $\overset{n}{\underset{\alpha =1}{\sum }}\Delta \gamma_{t}^{\left(\alpha \right)}$ represents the shear strain produced by all slip systems at time *t*.

**Figure 12:**
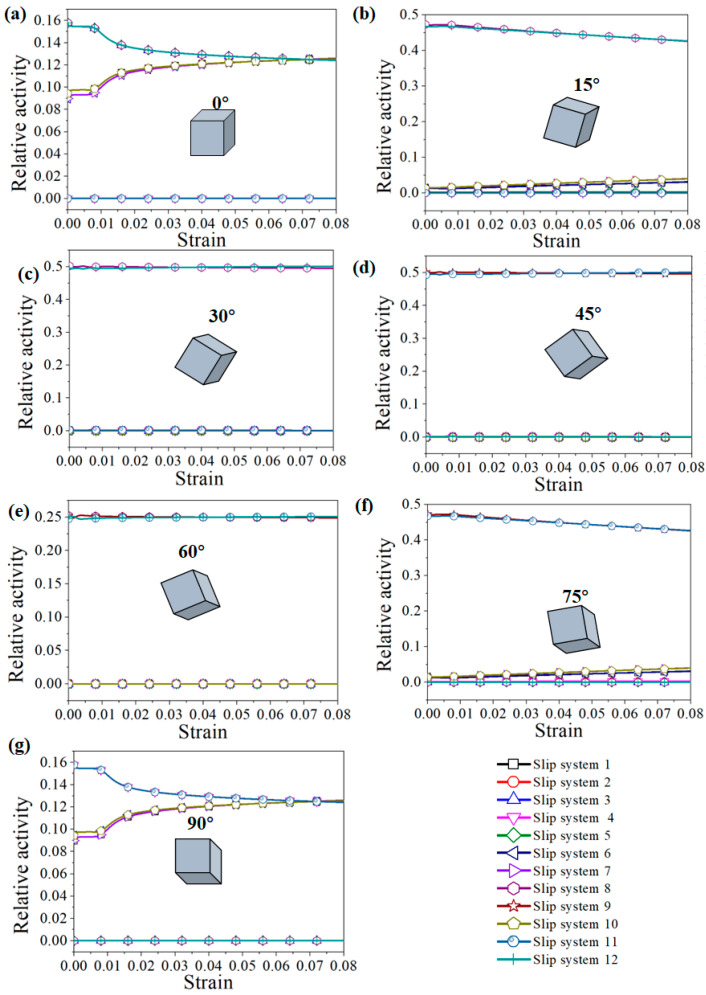
Activation of the slip systems of grains with different orientations during the tension process: (**a**) 0°, (**b**) 15°, (**c**) 30°, (**d**) 45°, (**e**) 60°, (**f**) 75°, and (**g**) 90°.

The relative activity of the slip system during the tension process of each grain is shown in Figure 12. Eight slip systems were activated in the 0° and 90° grains. With the increase in strain, the proportion of slip systems gradually increased, indicating that the contribution of the eight slip systems to the total shear strain was almost the same. For the 15° and 75° oriented grains, the shear strain of slip systems 8 and 12 accounted for 80% of the total shear strain, and the contribution of the other slip systems was only 20%. For the 30° and 60° grains, only two slip systems (slip system 9 and slip system 11) were activated, and the relative activity of the slip systems remained unchanged during tensile deformation. For the 45° oriented grains, there were four slip systems activated (slip systems 5, 6, 7, and 9), and the relative activity remained unchanged. It can be seen from the above analysis that when multiple slip systems were activated, the grain deformation was more uniform.

### 4.2. Effect of Misorientation Angle on Deformation Mechanism of Columnar Grains

As shown in Figure 7, the misorientation angles had a great influence on the stress distribution near the grain boundary. According to the principles of crystal plasticity and the crystallographic deformation of materials, when the misorientation angle is large, dislocations easily accumulate in the vicinity of the grain boundary, resulting in a stress concentration. It is necessary to activate a new dislocation source on the other side of the grain boundary to release the internal stress. For example, almost all slip lines of CG pure copper can pass through the low-angle grain boundaries without changing direction during the tensile process, resulting in a super-ductile deformation capacity [30,31]. For high-angle grain boundaries, due to the high degree of atomic mismatch at the grain boundary, the deformation coordination of both sides of the grain is poor.

**Figure 13:**
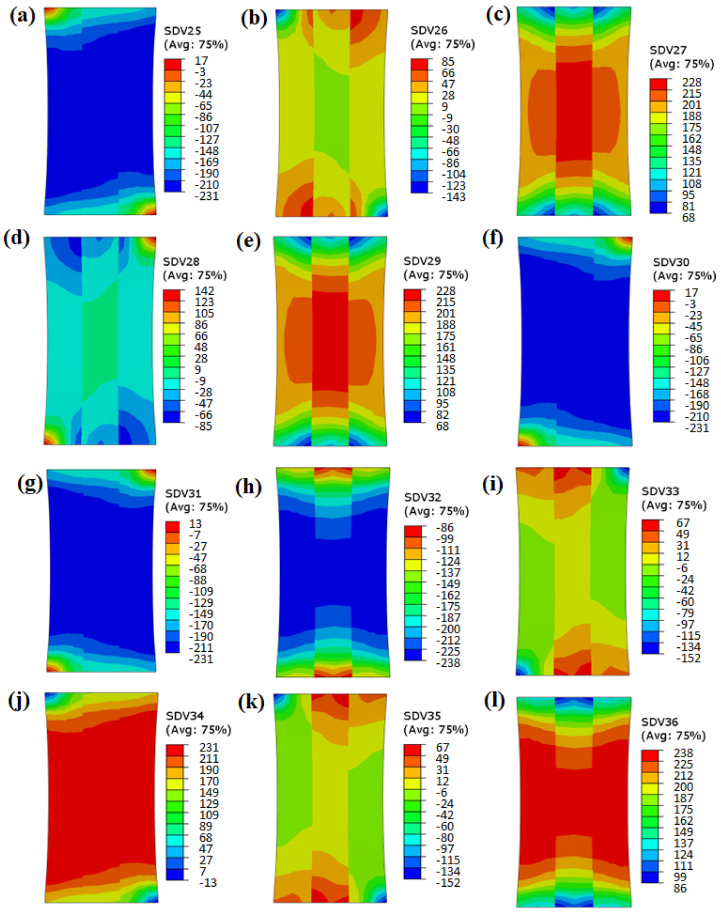
Shear stress of slip systems 1–12 with misorientation angle of 5° (SDV 25–SDV 36).

**Figure 14:**
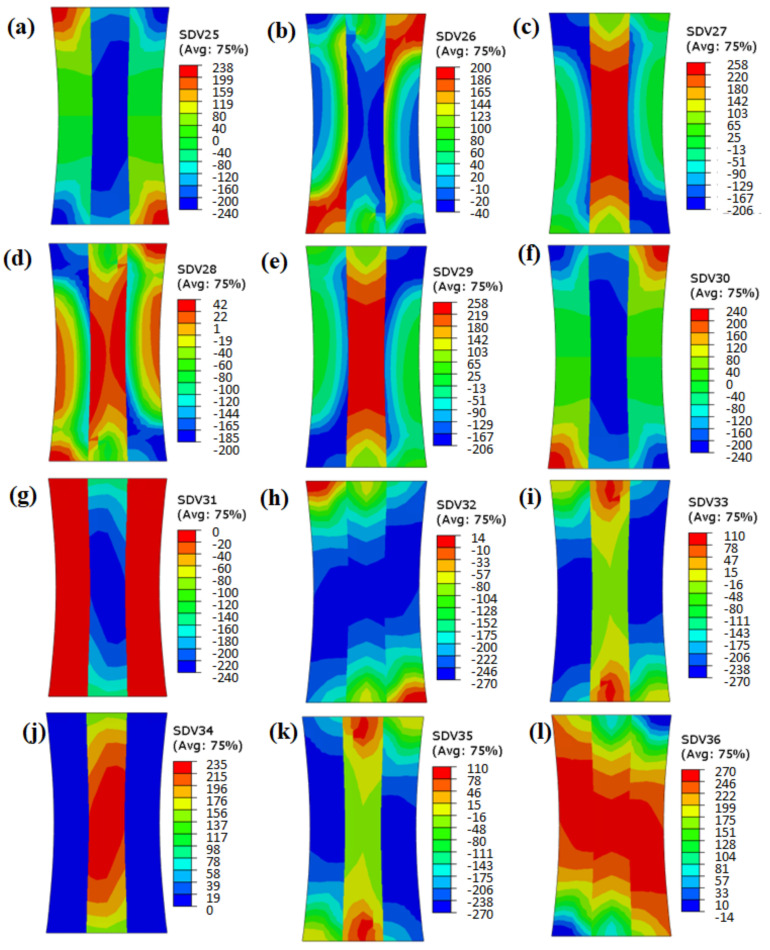
Shear stress of slip systems 1–12 with misorientation angle of 45° (SDV 25–SDV 36).

Figure 13 and Figure 14 show the shear stress values of the twelve slip systems (SDV 25-SDV 36) with misorientation angles of 5° and 45°, respectively. It can be seen from Figure 13 that the shear stress value of the slip system was evenly distributed (indicated by the similar colors) in the grains with misorientation angles of 5°. There was no obvious sudden change in the stress value near the grain boundary, indicating that the activation and hardening of the slip systems were similar during the deformation process, without obvious deformation disharmony.

From the shear stress results of the grains with a misorientation angle of 45° (Figure 14), it is clear that the deformation behavior of the slip systems differed substantially. Only the distribution of shear stress of slip system 8 and slip system 12 was relatively uniform. The shear stress distribution of the other ten slip systems showed obvious discontinuity. When the misorientation angle was large, the differences in the shear stresses of the slip systems were huge. Finally, a stress concentration area formed in the vicinity of grain boundaries with high misorientation angles.

### 4.3. Effect of Grain Boundary Morphology on Deformation Mechanism

**Figure 15:**
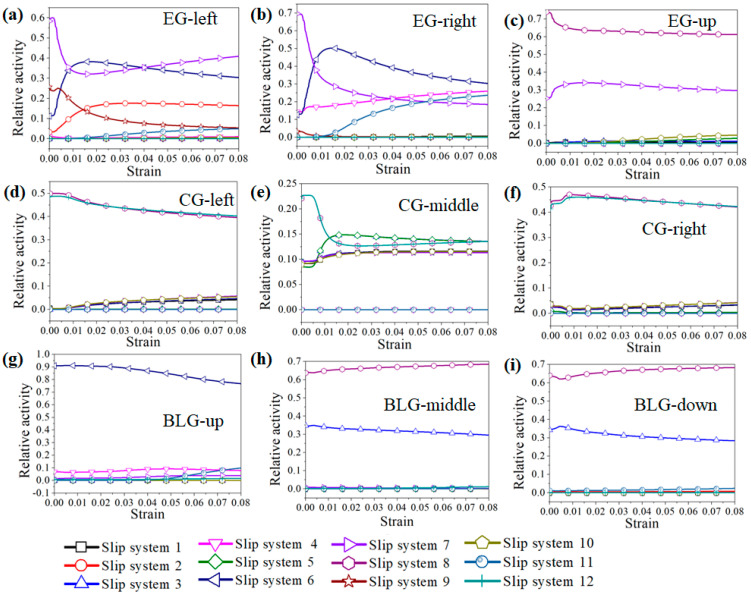
Activation of slip systems for different grain boundary morphologies: (**a**–**c**) triple-junction GB of EGs; (**d**–**f**) parallel GB of CGs; (**g**–**i**) transverse GB of BLGs.

Figure 9 and Figure 10 showed that the local stress results of different grain boundary morphologies were quite distinct, with obvious stress concentrations occurring near the triple-junction GBs of the EGs, while the stress distribution near the parallel GBs of the CGs and the transverse GBs of the BLGs was relatively uniform. The activation of the slip systems of the three grains near these characteristic grain boundaries were individually examined, as shown in Figure 15. From Figure 15a–c, it is evident that five main slip systems were activated in the left grain (EG-left) of the triple-junction GB, as well as the right grain (EG-right), but the number of activated slip systems in the upper grain (EG-up) was only two. Figure 15d–f shows that the number of activated slip systems in all the three grains near the parallel grain boundary of the CG was five. Meanwhile, the number of activated slip systems in the three grains near the transverse GB of the BLG (Figure 15g–i) was also five.

There are five independent constraints on the grain boundaries of ordinary polycrystals during deformation. Complex grain boundary deformation can be coordinated only by activating five independent slip systems. When the number of activated independent slip systems of the alloy is less than five, polycrystals experience incongruous grain boundary deformation, which easily leads to stress concentration and even cracking along the grain boundary. When the grain boundary has a straight morphology, such as in BLGs or CGs, the constraint conditions at the grain boundary during deformation are reduced to three, that is, only three slip systems need to be activated to ensure good deformation coordination. Similarly, for stress-induced phase transformation materials, a straight grain boundary morphology is conducive to the coordinated deformation and stress-induced phase transformation of the grains on both sides of the grain boundary and reduces the possibility of intergranular cracking due to stress concentration around the grain boundary. Ueland et al. [7] studied the similarities and differences between the stress-induced phase transformation of a Cu-Zn-Al alloy at the straight grain boundary and the triple-junction GB by in situ SEM experiments. It was found that the straight grain boundary could improve the shape memory performance of the alloy compared to the triple-junction GB, which was prone to stress concentration.

## 5. Conclusions

The crystal plastic finite element model (CPFEM) was used to analyze the influence of crystallographic orientations, misorientation angles, and grain boundary morphologies on the local stress distribution of a Cu-8Al-11Mn alloy subject to tensile deformation, and the deformation mechanism of local stress formation was revealed. The specific conclusions are as follows:

(1) The Schmid factor and number of activated slip systems played an important role in the stress value and distribution of single crystals. The effective stresses in the 0 ° and 90 ° grains with a high number of activated slip systems were smaller and more uniform than in the 15°, 30°, 45°, 60°, and 75° grains.
(2) When the grain boundaries of columnar grains had low misorientation angles, such as 5~15°, the local stresses in their vicinity were smaller and more uniform, with small difference in the shear stresses of the activated slip systems between the adjacent grains. However, the local stresses in the vicinity of the grain boundaries with high misorientation angles, such as 30~60°, increased sharply due to the large difference between adjacent grains in terms of the shear stresses of the activated slip systems.
(3) The grain boundary morphologies had a great influence on the local stress distribution of the polycrystalline grains. The local stress of the triple-junction GBs in the EGs was the largest, while that of the transverse GBs in the BLGs was the smallest, which was mainly related to the number of activated slip systems in the vicinity of the grain boundaries.

## Figures and Tables

**Table 1:** Slip systems of Cu-8Al-11Mn alloy (wt.%).

Slip Plane	(111)			$(\bar{1}$11)			$\left(\bar{1}\bar{1}1\right)$			$(1\bar{1}$1)		
Slip direction	$[0\bar{1}$1]	$[10\bar{1}$]	$[\bar{1}$10]	[101]	[110]	$[0\bar{1}$1]	[011]	[110]	$[10\bar{1}$]	[011]	[101]	$[\bar{1}$10]

**Table 2:** Material parameters of Cu-8Al-11Mn alloy (wt.%).

Slip System	*h*_0_ (MPa)	*τ*_0_ (MPa)	*τ_s_* (MPa)	*n*
{111} <110>	200.8	110.8	200.8	10

## Data Availability

Not applicable.
